# Observation of Single Nanoparticle Collisions with Green Synthesized Pt, Au, and Ag Nanoparticles Using Electrocatalytic Signal Amplification Method

**DOI:** 10.3390/nano9121695

**Published:** 2019-11-27

**Authors:** Sasikala Sundar, Ki Jun Kim, Seong Jung Kwon

**Affiliations:** Department of Chemistry, Konkuk University, 120 Neungdong-ro, Gwangjin-gu, Seoul 05029, Korea; sasikala412@gmail.com (S.S.); kim573252@naver.com (K.J.K.)

**Keywords:** biosynthesis, single nanoparticle, collision, Pt nanoparticle, Au nanoparticle, Ag nanoparticle

## Abstract

This work describes the tailored design, green synthesis and characterization of noble metal (Pt, Ag and Au) nanoparticles (NPs) using Sapinduss Mukkorossi fruit extract (SMFE) and its signal NP collision signal response, based on the principle of the electrocatatlytic amplication (EA) method. Here, the SMFE can act as both the reducing and the capping agent for the fabrication of noble nanometals. The SMFE-capped NPs was available for the observation of a single NP collision signal. Two general types of current response were observed: a staircase current response for the Pt or Au NPs, and a blip/spike current response for Ag NPs. These results demonstrated that the eco-friendly synthesized SMFE-capped NPs maintained their electrocatalytic activity, therefore they can be used for the single NP experiments and place an arena for future biosensing applications.

## 1. Introduction

With the growing need to minimize or eliminate the use of environmental-risk substances, as the green chemistry principles describe, the synthesis of noble metal nanoparticles (NPs) using biological entities has received increasing attention in the last decade [1]. Due to their unique physical and chemical properties, metal NPs have attracted tremendous interest in modern chemical research, and have found applications in a wide variety of fields, such as photochemistry, electrochemistry, optics and catalysis. To gain a better understanding of their fundamental properties and to optimize their activity for various applications, NPs must be characterized precisely in terms of size, shape and composition [2]. For example, both the size and shape of NPs have been shown to affect their catalytic activity. Biosynthesis of metal NPs has gained immense interest towards researchers, and it acts as a suitable substitute for hazardous chemical methods to achieve nanostructured materials with the controlled morphology of shape and size [3,4,5,6].

By the involvement of metal NPs, single NP collision experiments have played a vital role due to the rapidity, simplicity and extra power permitting for the fast investigation of thousands of NPs in seconds/minutes [7,8,9,10,11]. Methods to detect a single NP in a solution are generally referred to as the NP‘s “collisions” or “impacts” with the ultramicroelectrodes (UMEs) surface. The UME is a working electrode with the geometric dimensions of micrometer, to provide the means to contact with individual NPs suspended in the solution.

Bard and others reported several electrochemical approaches for detecting single NPs by measuring their impact with the conducting surface of the UME [12,13,14,15,16,17,18]. For example, Bard and coworkers observed a large current amplification due to electrocatalytic processes (oxidation/reduction of a species present in the electrolyte solution) occurring on the surface of an NP when it collided with the inert UME that otherwise could not electrocatalyze the reaction [12,13,14,15]. Compton and coworkers immobilized redox-active *p*-nitrophenol ligands on the metal NPs and monitored the current transients corresponding to the reduction of the attached ligands whenever the NPs contacted the electrode that was held at a potential negative enough to reduce the *p*-nitrophenol. These methods attempted to quantify the current transients to determine NP size distributions, but there are certain aspects associated with the chronoamperometric (CA) response that are not fully understood. Two types of CA responses have been observed: a current “step” as a result of NP accumulation, and a current “spike” or “blip” as a result of NP deactiviation or decomposition. The type of NPs and the electrocatalytic reaction can also influence the shape and magnitude of the CA response of the individual NP‘s collisions [16]. For instance, the citrate-capped Pt NPs on the Au UME showed the stepwise current increase (staircase response) for the hydrazine (N_2_H_4_) oxidation reaction, but the IrO*_x_* NPs on the Pt UME showed a spike (blip) response for the water oxidation reaction. In order to achieve those types of collision signal, each form should be categorized by the strategy for NP detection, which is defined by the selection of three major materials/reagents necessary for achieving single-particle detection: (1) the electrode material, (2) the colloid (i.e., NP) and (3) the electroactive redox molecules in solution that are oxidized or reduced upon NPs/UME impact to generate an observable electrochemical signal [19].

The electrocatalytic activity of NPs is varied depending on many variables, such as the surface facet or the capping agent. The capping agent of the NP determines the shape or size of the NP depending on the type and concentration of it. In the field of electrocatalyst, it is well knwon that the use of bulky or strongly-adsorptive capping agent, for example, poly-ionic polymers or thiols, will reduce the electrocatalytic activity of the NP by preventing the approach of reagent onto the surface of this same NP [20,21]. Therefore, only limited capping agents such as citrate or ascorbic acid are used yet for the single NP collision experiment, because the single NP collision is based on the high electro-catalytic activity of the NP. In the previous most of research, the citrate-assisted noble metal NPs have been used for the single NP collision experiments [21,22,23,24,25,26,27,28].

In this study, we have prepared three different types of NPs, like Pt, Au and Ag NPs, by using a novel greener surfactant, Sapinduss Mukkorossi fruit extract (SMFE). The greener surfactants in the SMFE consist of six major types of saponin molecules with a big polar hydrophilic head and long chain hydrocarbon [27]. This is a rapid proficient, inexpensive and environment-friendly bio-surfactant-assisted approach for the preparation of NPs [29,30]. Then, the prepared NPs were used for the single NP collision experiments using the EA method with the hydrzaine oxidation as the electrocatalytic indicator reaction. The current response for the single NP collision was successively observed with the SMFE-capped NPs, unlike the strongly adsorptive material-capped NPs. To the best of our knowledge for the first time, we have reported single NP collision measurements with the natural greener surfactant, SMFE.

## 2. Materials and Methods

### 2.1. Reagent

All chemicals were the analytical grade and used as received. Sodium phosphate dibasic anhydrous (Na_2_HPO_4_), sodium phosphate monobasic (NaH_2_PO_4_), hydrazine solution (N_2_H_4_) (35%), silver nitrate (AgNO_3_), hydrogen tetrachloroaurate (III) trihydrate (HAuCl_4_), and hexachloroplatinic acid solution (H_2_PtCl_6_) (8%) were purchased from the Sigma-Aldrich (St. Louis, MO, USA). All chemicals were used as received, unless otherwise stated. Ultrapure water (>18 MΩ, Millipore, Darmstadt, Germany) was used in all experiments.

### 2.2. Preparation of Sapinduss Mukkorossi Fruit Extract (SMFE)

About 10 g of the dried Sapinduss Mukkorossi fruits were dissolved in 100 mL water and stirred overnight by a magnetic stirrer. Then the mixture was filtered through Whatman No. 1 filter paper, and the filtrate was stored at 4 °C and used for the further experimental process [30].

### 2.3. Preparation of NPs and Characterization

Pt NP was prepared by the reduction of H_2_PtCl_6_ using SMFE as a reducing/capping agent. About 30.7 µL (0.5 mM) of H_2_PtCl_6_ was dissolved in 10 mL of water and allowed to stir for 10 min. After that, 5 µL of 0.05% *v*/*v* SMFE extract was added dropwise into the above mixture. The bio-reduction of H_2_PtCl_6_ was confirmed by the conversion of yellow color into brownish-black color colloidal sample and it also permitted to stir for 30 min.

To synthesize the Au NP, 3 µL of 0.06% (*v*/*v*) SMFE was added to 5 mL of 0.3 mM HAuCl_4_ aqueous solution. The bio-reduction of the HAuCl_4_ is confirmed by the slow color change from yellow to stable pink color after 8 h.

In the case of Ag NP, about 0.5 mM of AgNO_3_ was prepared with 25 mL of water. After then, 2 mL of 8% (*v*/*v*) SMFE extract was added dropwise into the AgNO_3_ solution at 80 °C. When the reaction time was increased, the color of the solution gradually changed from gray to brown, which indicated the reduction of Ag^+^ ions. Then, the solution was stirred for 1 h. Obviously, no additional reducing agent or surfactants were needed for the synthesis of these Pt, Au and Ag NPs.

The formations the of Au and Ag NPs were monitored under UV-Visible spectrophotometer (Carry 300 UV-Vis, Agilent, Santa Clara, CA, USA) by withdrawing aliquots of 2 mL at regular time intervals at a wavelength range of 200–800 nm. The morphology and size of the particles were examined using dynamic light scattering (DLS) (Zetasizer Nano ZS90, Malvern, Worcestershire, UK) and TEM (Tecnai G2 F30ST, FEI Company, Hillsboro, OR, USA). Samples for TEM studies were prepared by placing a drop of the Au, Ag or Pt NPs‘ colloidal suspension obtained by bioreduction on lacy carbon-coated grids. The zeta-potentials of the NPs were also measured by Zetasizer Nano ZS90. The electrochemical experiments were performed using a CHI model 660 potentiostat (CH Instruments, Austin, TX, USA) equipped with a three-electrode cell placed in a Faraday cage. The electrochemical cell comprised an UME (Au, Pt, or C-fiber) as a working electrode, a Pt wire counter electrode and an Ag/AgCl (1 M KCl) reference electrode.

### 2.4. Preparation of UME

A Carbon fiber (C-fiber) UME of 11 μm diameter was purchased from BASi (West Lafayette, IN, USA). Au and Pt UME were prepared using the general procedure used in our lab. Au and Pt UMEs were fabricated by heat-sealing 10 µm diameter metal wire (Goodfellow, Huntingdon, England, UK) in borosilicate glass capillaries (2.0 mmOD; 1.16 mmID, Sutter Instrument) under vacuum. Polishing paper of 800 and 2200 grit was used in exposing the metal surface, followed by polishing with 1 μm, 0.3 μm and 0.05 μm alumina (Buehler, Lake Bluff, IL, USA).

### 2.5. Electrochemical Cell

The solution of the electrochemical cell contains 15 mM hydrazine in 50 mM phosphate buffer (PBS). Cyclic voltammetry (CV) was performed at a 50 mV/s of scan rate in a pH 7.4 (alkali) phosphate buffer solution. Prior to every collision experiment, the Au and C-fiber UME was scanned over the potential range from −0.4 to 0.4 V (vs. Ag/AgCl) until a stable voltammogram was obtained. In order to observe the NP collision signal, the Au UME was held at an appropriate potential, with a data acquisition time of 50 ms. 

## 3. Results & Discussion

The synthesized NPs were characterized using the various methods below.

### 3.1. UV-Visible Spectroscopic Measurements

**Figure 1:**
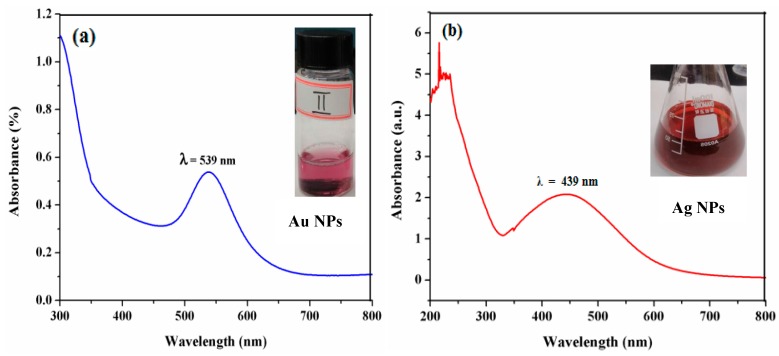
UV–visible spectra of (**a**) Au NPs and (**b**) Ag NPs synthesized using SMFE.

The UV–Vis spectroscopy is one of the most common techniques for authentication of the formation and stability of NPs in the aqueous solution. The bioreduction of Au(III) and Ag(I) by using SMFE have been confirmed through UV−visible measurements and carried out to identify the formation of Au and Ag NPs measured in the spectral range of 200–800 nm (Figure 1). Au NPs have been reported to exhibit a dark purple color in aqueous solution that is related to their intensity and size, owing to its surface plasmon resonance (SPR). The spectral analysis revealed that the SPR absorption maxima peak of Au NPs occurred at 560 nm with a high absorbance value [31], which is specific for Au NPs (Figure 1a).

Ag particles at the nano-range exhibit an unusual optical phenomenon called SPR, due to the cumulative oscillation of the conducting metal surface electrons in resonance with the non-particulate radiation. This property is largely governed and dependent upon the particle type, size, shape and the local chemical ambience. The characteristic fingerprint zone which exhibits this phenomenon predominantly appears in the range of 400–500 nm, respectively. The absorption spectra of the Ag NPs synthesized using SMFE are shown in Figure 1b. The UV–visible spectrum shows the formation of Ag NPs, as the peak maxima at 442 nm is the characteristic absorption for the Ag NPs [32,33].

### 3.2. Dynamic Light Scattering (DLS) and Transmission Electron Microscope (TEM) Measurements

**Figure 2:**
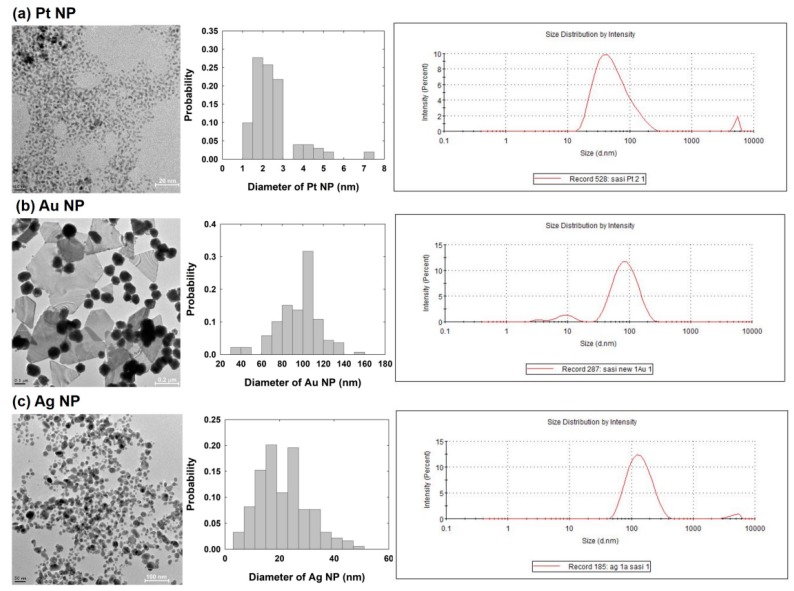
Transmission electron microscopy (TEM) images, size distribution and DLS measurement of (**a**) Pt NPs, (**b**) Au NPs and (**c**) Ag NPs synthesized using the SMFE.

The size and shape of the NPs synthesized using various content of the SMFE were clearly examined using TEM at an operation voltage of 80 kV. Figure 2 shows the TEM images of the Pt, Au and Ag NPs.

Figure 2a demonstrated the TEM image of Pt NPs, and the NPs are mono-dispersed and spherical in shape, with an average particle size of 2.6 ± 1.2 nm. Figure 2b shows that the Au NPs exhibited various shapes, including quasi-spheres or flat-planes. The spherical Au NPs have an average diameter of 95 ± 20 nm. The TEM image of the Ag NPs showed a typical spherical morphology with a homogenous dispersion of particles (Figure 2c). The average diameter of Ag NPs was found to be 21 ± 10 nm. All of the sizes of the NPs were obtained based on the TEM image. The DLS measurements are also shown in Figure 2. The measured size by DLS reveals relatively larger values than that obtained by TEM. The discrepancy between DLS and TEM measurements seems owing to the aggregation of NPs and/or the difference between hydrodynamic environment and dried condition of the NP. The zeta-potentials of the Pt, Au and Ag NPs were −31.8, −25.9, and −13.0 mV, respectively.

### 3.3. Single NP Collision Experiment

**Figure 3:**
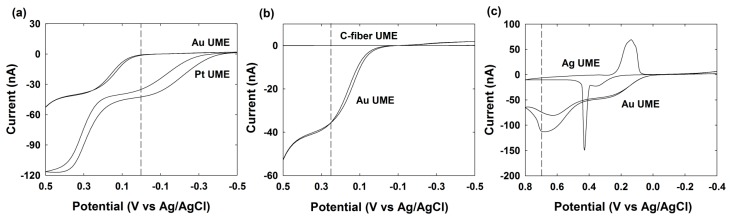
Cyclic voltammogram of hydrazine oxidation at (**a**) Au UME and Pt UME, (**b**) C-Fiber UME and Au UME, and (**c**) Ag UME and Au UME in 50 mM phosphate buffer (PBS) (pH 7.4) containing 15 mM hydrazine at 50 mV/s of scan rate.

First, the electrocatalytic activities of Pt, Au, Ag and C-fiber UME for the hydrazine oxidation reaction were tested by CV to determine the appropriate potential windows for the single NP collision experiments (Figure 3). Here, the appropriate potential is where the designed electrocatalytic reaction has occurred only at the NP, and not the UME. For the detection of the Pt NP, the 0 V of applied Au UME is adaptable for the NP collision observation. The C-fiber UME showed worse electrocatalytic behavior than the Au UME in the given potential region, resulting in a low background current; therefore, the C-Fiber UME at 0.25 V was selected for Au NPs collision experiments. In the case of Ag NP, different collision behaviors were observed depending on the applied potential [34]. In this study, the Au UME at 0.7 V was selected for the best distinguishing of the collision signal.

In the following sections, we have investigated the electrocatalytic activity of SMFE-capped Pt, Au, or Ag NPs by monitoring the hydrazine oxidation current generated in the event of single NP collisions using CA.

### 3.4. Pt NPs Collision Signal on Au UME

**Figure 4:**
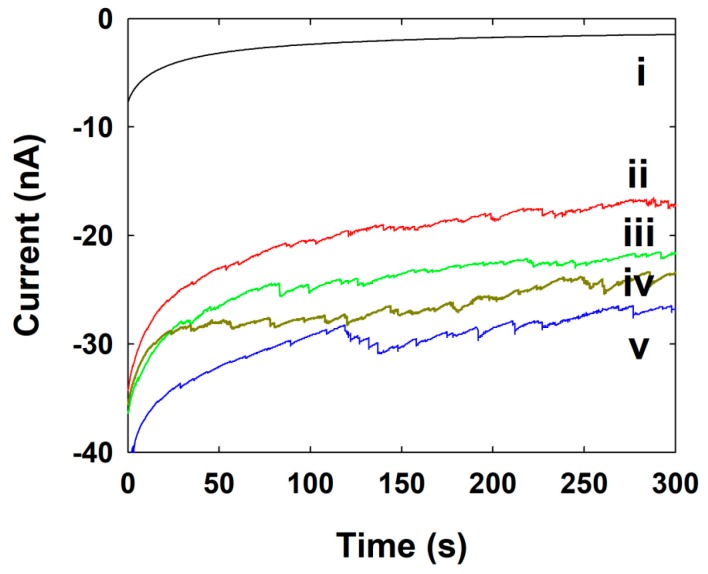
CA curves for single Pt NPs collisions at the Au UME in pH 7.4 buffer solution (15 mM hydrazine) containing various concentrations of Pt NPs (i) 0, (ii) 0.86, (iii) 1.70, (iv) 2.60, and (v) 3.40 nM at 0 V of applied potential. Data acquisition time is 50 ms.

The current signals by a single Pt NP collision were observed using CA measurement. The Pt NP was injected into the electrolyte solution containing the specific potential (0 V for here) applied Au UME. As shown in the Figure 4, the oxidation current or frequency gradually increased after injecting the various concentrations of Pt NP solution into the 50 mM phosphate buffer (pH ~7.4) containing 15 mM hydrazine. Usually, the oxidation of hydrazine does not occur at Au UME electrodes at this potential around 0 V, but it occurs at significantly higher rates of Pt at this potential, as shown in Figure 3a. Therefore, when the individual Pt NPs adsorbed on an Au UME, hydrazine oxidation is turned on, resulting in independent corresponding current transients. In this case, we have noted that the current increased in a stepwise fashion, signaling the individual adsorption events of Pt NPs. Since the staircase response suggested that the NPs stick to the electrode upon contact, the further collisions contribute to the accumulative increase of overall current [12,14].

In this case, the signal-to-noise (S/N) ratio changes with time and each new collision event, thereby making it difficult to detect individual NP collisions over long analysis times, and to determine whether other processes are involved, such as surface-induced NP aggregation or electrode fouling [28]. The basic principle involved in electrochemical methods (single NP collision experiments) for detecting NPs is to hold the potential where the electrode cannot catalyze a certain reaction, whereas the NP is capable of this upon its impact with the electrode. Therefore, the applied potentials at which Au and Pt catalyze the reaction play a key role in observing single NP current transients. In the case of an Au UME, Pt NPs remain catalytically active after sticking to the Au electrode [35]. Hence, after a certain time there is a buildup of electrocatalytic Pt NPs on the surface of the Au UME. Since Pt NPs stuck to the Au UME are capable of catalyzing hydrazine oxidation, the onset potential for hydrazine oxidation at Au UME shifts to a more negative value. In an amperometry curve, the current in each individual transient was somewhat different, indicative of the variable size or activity of individual Pt NPs adsorbed at the Au electrode surface. The frequencies of NP collisions have also been controlled by the concentration of Pt NP. When the concentration is sufficiently low (a few nanomolar), collision of single particles one at a time could be observed as random current transients. The amplitude of each current is a function of the particle size and the kinetics of electrocatalytic reaction at the particle surfaces. In general, the currents observed at various electrode potentials can be used to determine the kinetics for single Pt NPs [34,36], but in this case, we only compare the frequency of the particles at the same electrode potential among the different concentrations of Pt NPs.

When the NP that can catalyze the inner sphere reaction at that same potential collides and sticks to the UME surface, it behaves as a nanoelectrode and produces a step increase in the faradaic current relative to that of the UME. In such NP collision experiments, one can learn a lot from the collision frequency and from the height and shape of the faradaic response. Because the collision of the NP onto a UME is stochastic (random), diffusion-limited mass transfer to the electrode is represented by an average collision frequency [35,37] given by the equation below: [25]

(1) $$f_{NP}=4k_{ads}D_{NP}C_{NP}r_{UME}$$

where *f_NP_* is the collision frequency governed by diffusion of the NP to the UME, *K_ads_* is adsorption co-efficient of NP for the electrode surface (considered as 1 in here), *D_NP_* is the diffusion coefficient of the NP, *C_NP_* is the NP concentration and *r_UME_* is the radius of the UME (5 µm). The diffusion coefficient of the Pt NPs, 1.90 × 10^−10^ m^2^/s, which is calculated by the following Stokes–Einstein equation:

(2) $$D_{NP}=k_{b}T/6\pi \eta r_{NP}$$

where *k_b_* is the Boltzmann constant (1.38 × 10^−23^ J/K), *T* is the temperature (298 K), *η* is the viscosity of water (8.99 × 10^−4^ g·m^−1^s^−1^) and *r_NP_* is the radius of the Pt NPs (1.28 nm).

The collision frequencies of the Pt NPs have also been investigated from Figure 4, and it is proportional to the concentration of the Pt NPs. The calculated and experimental values of frequency at various concentrations of Pt NPs are given in Table 1.

The lower frequency seems to the aggregation of Pt NPs in electrolyte solution, the loss of NPs by adherence to the cell wall or precipitate, loss of signal by noisy background current, or a lower adsorption coefficient between Pt NPs and the Au UME. The surface aggregation of the Pt NP in the presence of hydrazine is reported in the previous study [28]. 

We have also calculated the value of the diffusion coefficient of hydrazine (*D_Hz_*) (1.036 × 10^−9^ m^2^/s), which is calculated from the equation: [12,14]

(3) $$I_{SS,UME}=4nFD_{Hz}C_{Hz}r_{UME}$$

where *I_SS,UME_* is the steady state current by the Au UME or Pt UME, *n* is the number of electron transfer for hydrazine oxidation reaction (*n* = 4), *F* is Faraday’s constant (96485 C/mol), *C_Hz_* is the concentration of the hydrazine (15 mM) and *r_UME_* is the radius of the Au UME (5 µm).

Based on the *D_Hz_*, the height of the current step under mass transfer control is also given by the equation: [12,14]

(4) $$I_{SS,NP}=4\pi ln\left(2\right)nFD_{Hz}C_{Hz}r_{NP}$$

where *I_SS,NP_* is the steady-state current for the NP and *r_NP_* is the radius of the Pt NPs. As a result of above calculation, the theoretical steady-state current by single Pt NP was 26 pA. However, the experimentally-obtained current step was 120 ± 110 pA (Figure 4), which is one order of magnitude higher than the theoretical value. The higher steady-state current step of the NP is due to the contribution by aggregated NP. Even though the frequency and steady-state current of single NP collision is somewhat differing from the theoretical expectation, the single NP collision signal was successively observed using the SMFE-capped Pt NP. This is the first time for a single Pt NP collision observation using a specially capped Pt NP, excluding the citrate-capped Pt NP.

### 3.5. Au NPs Collision Signal on C-Fiber UME

The commercially-available C-fiber UME shows a low background current for hydrazine oxidation, and gives reasonable results for the electrocatalytic signal of Au NPs collision without any pretreatment of the UME or deformation of Au NPs [9]. The magnitude of the current transients produced by the Au NPs collisions and the collision frequencies were collected and compared to the size and concentration of the Au NP.

**Figure 5:**
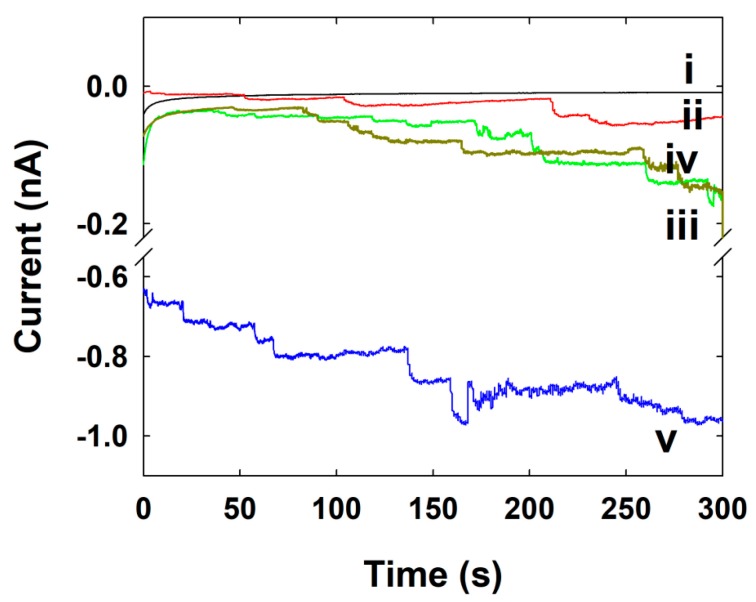
CA curves for single Au NPs collisions at the C-Fiber UME in pH 7.4 buffer solution (15 mM hydrazine) containing various concentrations of Au NPs (i) 0, (ii) 0.011, (iii) 0.023, (iv) 0.034, and (v) 0.045 pM at an applied potential of 0.25 V. Data acquisition time is 50 ms.

The C-fiber UME itself does not catalyze hydrazine oxidation at 0.25 V, as shown in the Figure 3b and Figure 5i. Therefore, we have not observed any current transient by the UME without NP. However, when the synthesized Au NPs were injected in to an electrolyte containing C-fiber UME at 0.25 V, the hydrazine oxidation is electrocatalyzed and the current transient was observed whenever the Au NPs collide with the C-fiber UME (Figure 5). The current increased in a stepwise manner through electrocatalytic amplification of Au NPs [9,26]. This typical behavior was observed at each and every time, once a small amount of Au NPs was injected into the solution containing hydrazine. The staircase response of the current signals was not uniform and shows some noisy signals, although some differences in shape and height were observed. This type of current transient most likely indicates the difference in the size and shape of the synthesized individual Au NPs.

The diffusion coefficient of the Au NPs was calculated as 5.10 × 10^−12^ cm^2^/s using the Equation (2). The collision frequencies of the Au NPs were also increased with the NP concentration, as shown in Figure 5.

The calculated and experimental frequency values of the Au NPs are given in the Table 2. The staircase response indicates that the collided Au NPs were stuck on the UME surface and the Au NPs were accumulated on the surface.

Using Equation (2), the theoretical steady-state current by single Au NP was calculated with 770 pA. However, the experimentally-obtained current step was 8.7 ± 9.4 pA (Figure 5), which is two orders of magnitude lower than the theoretical value. The higher collision frequency and lower steady-state current step of the Au NP indicate that the size of Au NP is smaller than our calculation. As shown in the Figure 2b, the synthesized SMFE-capped Au NP exhibited various shapes, including quasi-spheres or flat-planes. We have calculated the size of the NP and the concentration of stock solution, based only on the spherically-shaped one. Therefore, the plane Au nanostructure may contribute to the collision frequency, resulting in the higher frequency value. Details about the single nanostructure collision, such as plane shape, required further studies. In the previous result, the collision signal was disappeared with the thiol-capped Au NP [21]. Though the collision signal of Ag NP was very sensitive to the capping agents [9,21,26], the single NP collision signal was successively observed using the SMFE-capped Au NP.

### 3.6. Ag NPs Collision Signal on Au UME

**Figure 6:**
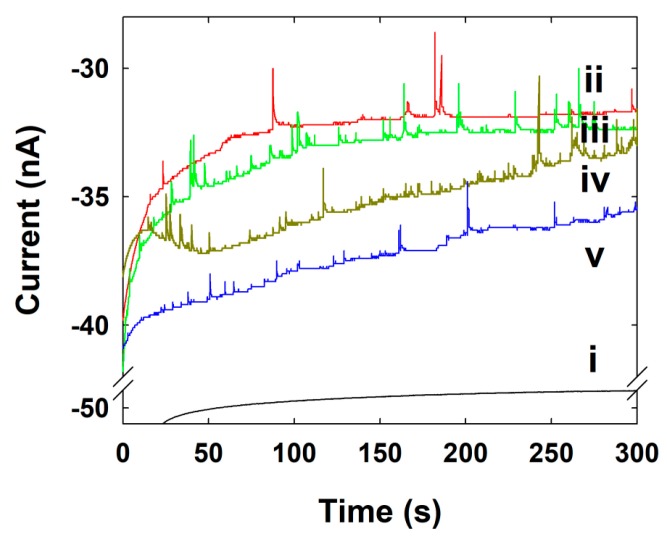
CA curves for single Ag NPs collisions at the Au UME in pH 7.4 buffer solution (15 mM hydrazine) containing various concentrations of Ag NPs (i) 0, (ii) 1.80, (iii) 3.60, (iv) 5.40, and (v) 7.20 pM at an applied potential of 0.7 V. Data acquisition time is 50 ms.

Figure 6 shows the CA trace recorded at Au UME held at a potential of 0.7 V in a 50 mM phosphate buffer (pH ~7.4) containing 15 mM hydrazine before and after loading with Ag NPs. The spike-shaped CA response was observed in the case of Ag oxidation with Ag NPs at an Au UME.

Previously, the single Ag NP collision signal was observed mostly based on the direct oxidation of Ag NP in the buffer solution [37]. However, in this study, the Ag NP collision was done in hydrazine solution. In the presence of hydrazine, the current response of Ag NP collision has various shapes, depending on the applied potential [34]. When the applied potential is lower than the Ag oxidation potential, the collided Ag NP blocks the electrocatalytic hydrazine oxidation reaction by the UME, resulting in a stepwise current decrease response. When the applied potential is higher than the Ag oxidation potential, the collided Ag NP undergoes oxidative dissolution on the UME, resulting in an inversed blip current response. In this study, the Au UME initially showed electrocatalytic current at 0.7 V. Therefore, when the Ag NP was collided on the Au UME surface, the electrocatalytic reaction by the Au UME was blocked by the collided Ag NP, resulting in instantaneous current decrease due to the hindered diffusion flux. However, at the same time, the oxidative dissolution of Ag NP occurred. Therefore, the electrocatalytic currents by Au UME are recovered [34]. Unlike the EA strategy, the single NP impact signals in the blocking strategy are thought to be not significantly affected by the NP’s capping agent. However, the collision of NP on UME and subsequent hindrance of the electrocatalytic reaction of UME by NP are closely related to the adsorption coefficient between the NP and UME. Therefore, the selection of the capping agent of NP can affect the collision signal by controlling adsorption strength between NP and UME. The whole process resulted in a spike-shaped current response, as shown in Figure 6.

In a CA curve recorded at the Au UME after the injection of Ag NPs, very distinct current spikes were observed. Normally, the current spike in a single collision showed a very fast decrease and a recovery, unlike the accumulative increase in current observed on Pt and Au UME. Most of the current spikes were quite uniform in magnitude, although there were some slight differences in the height and shape of some transients that we attribute to a polydispersity in the sizes and shapes of the particular nanoparticles, and small variations in the catalytic response and poisoning processes [8,38].

We have also observed that the frequency values of the Ag NPs increase with the increasing concentrations of Ag NPs in this experiment. The calculated and experimental values of the frequency of the Ag NPs at various concentrations of Ag NPs are given in Table 3. The diffusion coefficient of the Ag NPs, 2.33 × 10^−11^ m^2^/s, comes from the above Equation (2) where the 10.4 nm of the Ag NPs radius was used for the calculation of frequency.

The collision signal in the blocking strategy is obtained by the blocking of diffusive mass transfer of reagent from bulk to the UME by collided NP. Because the size of NP should be considerably large for the effective (or detectable) blocking, therefore not all the NP collision is reported as current signal response. These are also resulted in the lower collision frequency than the theoretical experctation, in addition to the aggregation effect.

As mentioned above, the choice of an appropriate surfactant is very important in the electrocatalytic activity of NP and the observation of the single NP collision signal. In this study, the single NP collision by SMFE-capped NP was investigated. Since the bio-assisted synthesis of NPs have their electrocatalytic activity of the NPs, the single NP collision signal was successfully observed in all three (Pt, Au, and Ag) types of NPs. However, the size of NPs differed considerably depending on the measuring method: DLS or TEM. Thus, the theoretically expected frequency based on the TEM measurement using the dried sample was in disagreement with the experimental results from the hydrodynamic condition. These effects in SMFE appear to be greater than the citrate case.

To investigate the influence of greener surfactant (SMFE) for the single NP collision signal, the experimental result was compared with the previous single NP collision experiments based on the citrate-capped NPs. As shown in the Table 4, mostly the peak height and frequency were decreased in SMFE-capped NPs than that of the citrate-capped NPs, except in the case of an unexpected high frequency of SMFE-capped Au NP. These results indicate that the SMFE has stronger binding affinity for the NP than the citrate.

## 4. Conclusions

In summary, a new biosynthesis has been demonstrated for the electrochemical detection of single NP collision events of the Pt, Au and Ag NPs onto the Au or C-Fiber UME. Since the electrical catalytic properties of a single NP are highly influenced by surfactants, the use of surfactant for the single NP collision experiments are limited. Therefore, the use of SMFE for the single NP collision can expand the application of this technique. We have observed the single NP events through the enhanced current by the electrocatalytic oxidation of hydrazine, when NPs contact the electrode and transiently stick to it. The overall current transient consists of repeated current spikes (Ag NPs) that return to the background level, superimposed on a current decay, rather than the staircase (Pt and Au NPs) response seen where an NP sticks onto the UME. 

Here the each event produces a unique current spike/blip or staircase. The frequency of the current transients was directly proportional to the concentrations of the NPs. A newer application of SMFE in the field of nanomaterials (Pt, Au and Ag) synthesis has been developed from both the economic and environmental points of view for the detection of a single NP collision signal.

## Figures and Tables

**Table 1:** Frequency values at various concentrations of Pt NPs.

Concentration of NPs (nM)	Calculated Frequency of NPs (s^−1^)	Experimental Frequency of NPs (s^−1^)
0.86	2.0 × 10^3^	0.50
1.70	3.9 × 10^3^	0.58
2.60	5.9 × 10^3^	0.63
3.40	7.8 × 10^3^	0.66

**Table 2:** Frequency values at various concentrations of Au NPs.

Concentration of NPs (pM)	Calculated Frequency of NPs (s^−1^)	Experimental Frequency of NPs (s^−1^)
0.011	7.0 × 10^−4^	0.04
0.023	1.4 × 10^−3^	0.11
0.034	2.1 × 10^−3^	0.18
0.045	2.8 × 10^−3^	0.20

**Table 3:** Frequency values at various concentrations of Ag NPs.

Concentration of NPs (pM)	Calculated Frequency of NPs (s^−1^)	Experimental Frequency of NPs (s^−1^)
1.80	0.51	0.19
3.60	1.02	0.44
5.40	1.53	0.59
7.20	2.04	0.46

**Table 4:** Frequency and peak height of SMFE- and citrate-capped NPs.

Surfactant	SMFE			Citrate		
NP	Pt	Au	Ag	Pt	Au	Ag
Diameter (nm)	2.6	95	21	4.3	18	30
Applied potential ^a^	0	0.25	0.7	0.1	0.6	0.8
Peak height ^b^	6.4	0.012	2.2	1.8	1.0	2.0
Frequency ^c^	5.8 × 10^−4^	3.6	0.10	0.017	0.010	1.7
Reference	This work	This work	This work	[14]	[26]	[34]

^a^ potential (V vs. Ag/Cl); ^b^ normalized peak height for the radius of NP and hydrazine concentration (pA·mM^−1^ nm^−1^); ^c^ normalized frequency (s^−1^ pM^−1^).
